# From resistance to reliance: A human-centered analysis of the spectrum of radiologists' trust in AI

**DOI:** 10.1016/j.ejro.2026.100780

**Published:** 2026-06-19

**Authors:** Kalina Chupetlovska, Eleni Georganta, Wouter Dignum, Saachi Yadav, Thi Dan Linh Nguyen-Kim, Regina Beets-Tan, Stefano Trebeschi

**Affiliations:** a Netherlands Cancer Institute, Amsterdam, the Netherlands; b University of Amsterdam, Amsterdam, the Netherlands; c City Hospital of Zurich, Zurich, Switzerland; d GROW - Research Institute for Oncology & Reproduction, Maastricht University, Maastricht, the Netherlands

**Keywords:** Radiology, Artificial Intelligence, Trust, Diffusion of Innovation, User-Centered Design

## Abstract

**Background:**

Artificial intelligence (AI) is increasingly applied in radiology, yet research has focused mostly on technical implementation, while human factors, particularly radiologists’ trust, which is critical for adoption, remain underexplored.

**Methods:**

We conducted semi-structured interviews with 18 radiologists from two hospitals using a guideline covering five domains: user, system, developer, ethical, and patient factors. Interviews allowed participants to elaborate, revisit points, contradict themselves, and introduce unanticipated topics. Thematic analysis with systematic coding identified key themes while preserving contextual nuance.

**Results:**

All participants had prior AI experience. Most (61%, 11/18) were optimistic about AI's potential for specific tasks. Accuracy/reliability (94%, 17/18) and time-saving (100%, 18/18) were consistently highlighted as the most critical factors for trust and adoption. Usability, including intuitive interfaces and seamless PACS integration, was emphasized by most (72%, 13/18), while half (50%, 9/18) noted the importance of transparency. Institutional reputation influenced trust in the large majority (89%, 16/18), with preference for non-commercial or reputable entities. Other important factors included clinician involvement in system design (56%, 10/18) and peer-reviewed evidence of performance (61%, 11/18). Ethical considerations included retaining human oversight (83%, 15/18), protecting patient privacy (44%, 8/18), and ensuring institutional data ownership (33%, 6/18).

**Conclusions:**

Successful integration of AI in radiology requires attention to radiologists’ perspectives and human factors throughout development, implementation, and use. The diversity and complexity of trust-related factors highlight the importance of human-centered approaches to AI adoption and the need for further research to guide effective implementation.

## 1 Introduction

Artificial intelligence (AI) is increasingly positioned as a transformative force in radiology, with the potential to improve diagnostic accuracy, automate time-consuming tasks, and help manage increasing imaging demands. However, despite rapid technical progress and a growing number of regulatory approvals, adoption in routine clinical practice remains uneven [1], [2]. A critical but underappreciated reason for this gap is trust. In high-stakes environments such as radiology, where decisions directly affect patient outcomes, the central question might not be whether AI outperforms radiologists, but whether radiologists are willing to trust AI and use its recommendations appropriately.

Trust is commonly defined as a psychological state comprising the intention to accept vulnerability based on positive expectations of another entity’s intentions or behavior [3], [4]. In AI contexts, this extends to human–machine interactions, where clinicians must decide whether, when, and how to rely on algorithmic outputs. Research consistently demonstrates that trust strongly predicts technology adoption [5], [6], making the understanding of its antecedents a central concern for researchers, developers, and policymakers [7]. In clinical settings, both over-reliance (automation bias) and under-reliance can negatively affect outcomes [8], [9], while AI's reshaping of cognitive task distribution raises further concerns about deskilling, liability, and accountability [10], [11].

Despite growing interest, our understanding of what shapes radiologists' trust in AI remains incomplete. Most studies rely on surveys that offer limited insight into the reasoning behind trust formation [1], [12], and existing qualitative work is narrow in scope — focusing on broad stakeholder groups rather than radiologists specifically [13], or on awareness rather than trust [14]. Existing frameworks tend to examine single dimensions such as transparency [15], [16] or technical performance [6] in isolation, and no study has yet applied a unified framework integrating user, system, developer, ethical, and patient-related factors specifically to radiologists.

To address this gap, we explore radiologists' trust in AI through semi-structured interviews with 18 radiologists with experience using AI in clinical or research settings, recruited from two European university hospitals. By focusing on professionals with direct experience of these systems, we gain nuanced insights into how radiologists evaluate, adopt, and respond to AI in their work environment. The analysis is guided by a framework building on dimensions from human–computer interaction research [17], extended with a multi-level view of trust within broader socio-technical systems [18], bringing together perspectives often treated separately to advance understanding of trust in clinical AI and offer practical guidance for its implementation.

## 2 Methods

### 2.1 Sample

**Fig. 1:**
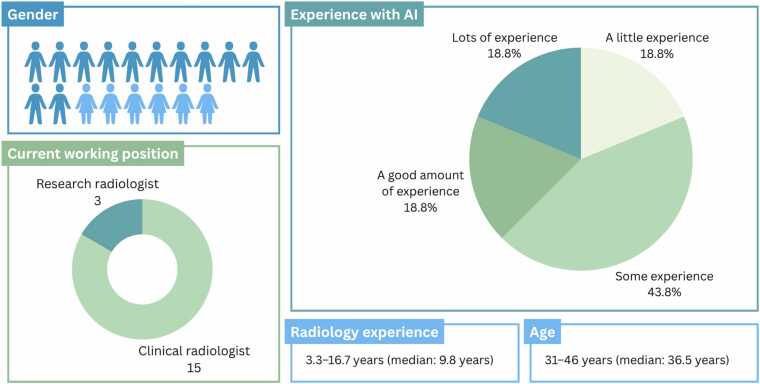
Demographic characteristics of the study participants. Data on age and years of experience were available for 16 of the 18 participants, as two did not provide this information.

Twenty-three radiologists were invited to participate across two university hospitals (Netherlands Cancer Institute, Amsterdam, The Netherlands and City Hospital of Zurich, Zurich, Switzerland), of whom 18 agreed, resulting in a non-participation rate of approximately 22%. All were based in the Netherlands or Switzerland, with the majority originally from these countries and a minority from Belgium, Cyprus, Germany, Luxembourg, and Ukraine, providing a degree of international diversity. Three participants were engaged in research, while the remainder worked in clinical practice (Fig. 1). Importantly, all had prior experience with AI in clinical or research settings. Participants had used or were using AI tools in clinical work, including lung nodule analysis, stroke perfusion assessment, pulmonary embolism detection, evaluating breast lesions on mammography, and detecting prostate lesions on MRI. In addition, some (39%, 7/18) were involved in AI training, testing, and developing algorithms for automated tumor detection and volume measurement. Both hospitals are located in countries where AI-supported diagnostic technologies are increasingly available and integrated into clinical workflows. The inclusion of both practitioners and researchers with direct AI experience allowed participants to reflect on trust from an informed perspective, capturing a range of supportive and critical viewpoints.

### 2.2 Study design

**Fig. 2:**
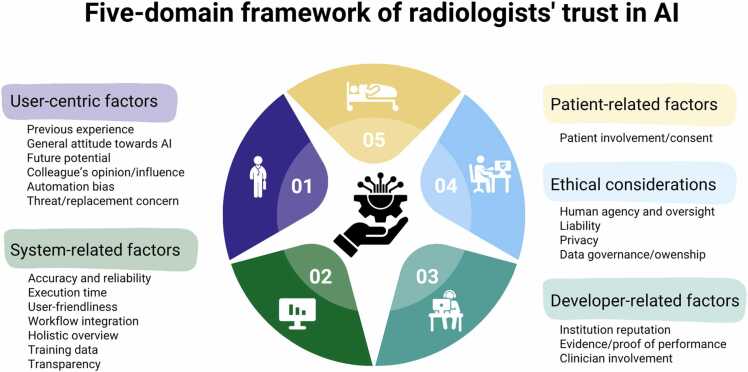
Overview of the five domains and key subthemes shaping radiologists' trust in AI.

Semi-structured interviews were chosen to explore trust in AI from a human-centric perspective, allowing the study to address predefined areas while accommodating unexpected perspectives. The Interview Guide was developed by two of the authors, one of whom has extensive expertise in qualitative interview methodology and trust in AI, based on a review of existing literature on trust in intelligent systems across medicine, human–AI interaction, and psychology. Identified factors were organized into five thematic domains: user factors (e.g., prior experience, attitudes, expectations), system-related characteristics (e.g., accuracy, transparency), developer-related considerations (e.g., reputation, clinician involvement, liability), ethical aspects (e.g., human oversight, data governance, privacy), and patient-related factors (e.g., patient trust in AI) (Fig. 2).

The guide was further refined through a pilot phase of three trial interviews conducted within the author team. In the first pilot interview, the more experienced interviewer led the session while the other two authors observed. In the second and third, one of the other authors conducted the interview while the experienced interviewer observed. This process informed refinements to question phrasing, structure, and flow, and resulted in standardized interviewer instructions to reduce potential interviewer bias. The final interview guide is provided in Supplementary Material (Appendix 1). Interviews were conducted until thematic saturation was reached, with no substantially new themes emerging in the final interviews.

### 2.3 Study procedure

Data collection commenced following approval from the Ethics Committee of University of Amsterdam, Amsterdam, The Netherlands. Participants were contacted via email and informed about the study's focus on trust in AI. Interviews were conducted in person or online, lasting a median of 30 min, and were anonymized with no identifiable information in recordings or transcripts. A brief demographic survey was administered separately via Qualtrics to prevent matching between demographics and interviewees. Interviews were conducted by three researchers, one research assistant, one expert in trust in AI, and one expert in radiology. To ensure a common understanding and consistent application of the interview guideline, initial interviews were conducted with all three researchers present, followed by a debriefing session to align on interpretation and approach. Following the pilot phase, the two trained interviewers conducted the remaining interviews independently, with each interview led by one interviewer. The semi-structured format allowed participants to revisit points, elaborate freely, and introduce new ideas beyond the guide's predefined topics.

### 2.4 Data analysis

All interviews were transcribed verbatim using Amberscript (Amberscript Global B.V., Amsterdam, the Netherlands; accessed June 2025; https://www.amberscript.com) and Otter AI (AISense Inc., Mountain View, CA, USA; accessed June 2025; https://otter.ai) and subsequently reviewed against the original audio recordings by a radiologist co-author to ensure accuracy, with particular attention to technical radiological terminology. Transcripts were analyzed using an iterative deductive–inductive thematic coding approach. Transcripts were first coded deductively using the predefined coding scheme, and subsequently enriched inductively to capture emerging themes not fully represented in the initial framework. Coding was primarily conducted by one author under the supervision of a senior author with expertise in qualitative analysis, with ongoing iterative discussions to refine interpretations and ensure consistency. The final thematic synthesis was completed by a senior member of the author team, integrating coded material into higher-order themes aligned with the five overarching domains (see final Codebook in Appendix 2). Representative quotes were selected to illustrate key themes, chosen on the basis of clarity and typicality, with divergent views also included where they added analytical depth. Full details of the methodological approach, reported in accordance with the Consolidated Criteria for Reporting Qualitative Research (COREQ), are provided in Appendix 3.

## 3 Results

All participants were asked the same standardized interview questions. Where frequencies are reported, counts indicate the number of participants (out of N = 18) who explicitly mentioned a given theme. These are reported for descriptive purposes only and do not represent statistical inference.

### 3.1 User-centric factors

**Fig. 3:**
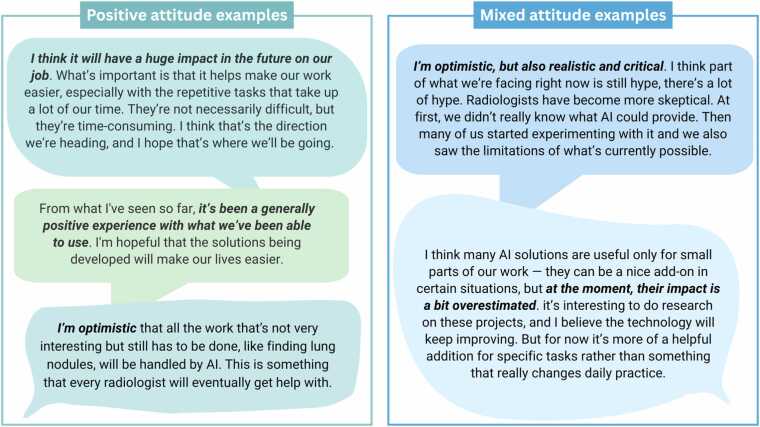
Selected quotes illustrating participants’ general attitudes toward AI.

Eleven (61%) participants described their general attitude toward AI as positive, five (28%) as mixed, and two (11%) did not specify. Notably, positive statements were often immediately followed by comments about future potential rather than AI's current state in radiology. Fourteen (78%) participants, including all those with mixed attitudes, believed AI has significant future potential. Mixed attitudes were typically framed as skepticism toward what AI can do today — seen as overhyped or limited to specific tasks — alongside optimism for future improvement. Selected quotes illustrating both attitude types are shown in Fig. 3. Additional quotations relating to all presented themes can be found in Supplementary Material, Appendix 4.

Ten (56%) participants reported that colleagues' opinions influenced their trust in AI, particularly positive experiences shared by respected peers or personally known colleagues.

Interestingly, only four (22%) participants mentioned concerns about radiologists losing their jobs to AI. Of these, two referred to it mainly in terms of increased efficiency rather than full replacement, e.g.:

“Probably each radiologist will be able to work faster with AI, so departments might need fewer radiologists overall. Some people could lose their jobs, or we might need to think about new roles or shifts in responsibilities for radiologists.” (Interviewee 2)

The other two expressed that AI could potentially replace humans, but only if it were to significantly improve its capabilities in the distant future:

“If AI were to write the entire report, review all the images, and produce something much more accurate than you could, you’d probably feel a bit threatened — wondering, what am I going to do now? But I think that scenario is still quite far in the future. For now, AI is more about doing specific tasks very well.” (Interviewee 6)

The majority of participants, however, doubted AI could ever fully substitute for human radiologists, citing a range of reasons (Table 1).

**Table 1:** Perceived barriers to AI replacing radiologists.

**Reason**	**Explanation**
*Limited to specific tasks*	AI excels at narrow, well-defined tasks but struggles with the breadth and complexity of full diagnostic workups
*Assistance over replacement*	AI is better suited to assist with simple, repetitive tasks (e.g., comparisons, measurements) than to manage complex diagnostic cases
*Human oversight remains essential*	Even highly accurate AI still requires humans to train, validate, and verify its outputs
*Risk of missing incidental findings*	Task-specific AI may overlook unexpected findings outside its scope (e.g., incidental rectal cancer on a prostate MRI)
*Clinical communication and context*	Radiologists remain essential for discussing findings with clinicians and patients and for placing results within the broader clinical picture

A more common concern, raised by 7 (39%) participants, was automation bias and AI dependency (cognitive offloading), typically framed as a future rather than current risk. Participants worried that highly accurate AI might lead radiologists to become overly reliant on its outputs, particularly when screening negative exams or working under time pressure. Two (11%) participants expressed concern that future residents who rely heavily on AI during training might fail to develop their own diagnostic confidence and intuition. As one participant explained:

“It’s a bit like being taught to drive using only automatic cars — you never learn how to drive manually. If you go through training always using AI support tools, you don’t develop the same gut feeling or sense of what’s normal and abnormal as you would after reviewing hundreds of cases as a primary human reader.” (Interviewee 9)

Interestingly, one (6%) participant stated the opposite view, suggesting that AI would make them more alert by motivating them to catch the system's mistakes.

### 3.2 System-related factors

**Fig. 4:**
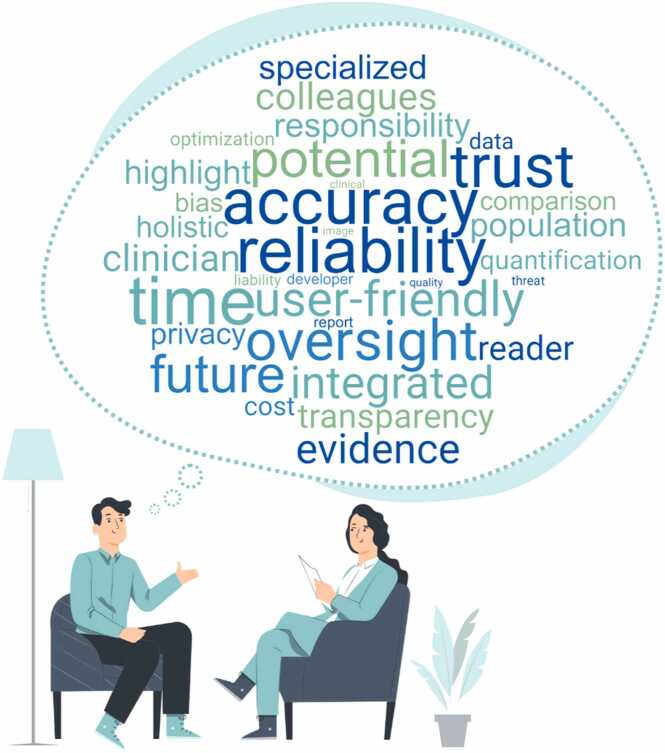
Word-cloud visualization of the most frequently mentioned factors.

All but one participant (94%, 17/18) identified accuracy and reliability as key factors influencing trust in AI, with most ranking them as the most important criteria (Fig. 4). Accuracy was described in terms of precision, low false positives and negatives, and high sensitivity and specificity, while reliability referred to consistency and robustness across different cases, scanners, and protocols. Three participants noted willingness to tolerate some false positives, provided they were not excessive and could be quickly verified, if the system was capable of catching findings a human might miss. A common example was AI flagging potential pulmonary embolism cases — occasional false positives were acceptable given the risk of missing a critical finding.

All participants (100%, 18/18) identified execution time of the AI program and potential time saving as key factors influencing their likelihood of using it. Most expressed a strong preference for AI outputs to be available instantly or already visible while reading the exam. They were reluctant to use a program that slows them down, for example, by generating too many false positives requiring manual checks or by presenting information in an inefficient or confusing way. However, a few (17%, 3/18) noted that they might be willing to sacrifice some time if it ultimately improved patient care, for example:

“In the end, it’s the consequences for the patients and the benefits for the patients that matter the most. I’m willing to sacrifice a little extra time as long as it’s helpful for the patient.” (Interviewee 18)

Conversely, some (11%, 2/18) participants emphasized that even very high accuracy would not justify using a program that significantly slowed them down:

“I think accuracy is the most important factor, as I mentioned. The second most important thing is that it saves time. Even if an AI is accurate, if it takes ten minutes per scan, you still wouldn’t use it.” (Interviewee 5)

“That’s great if the AI is very accurate, but if it ends up taking longer to get the results into the report, checking the software, loading the images, and so on, it might take eight minutes in total. In that case, I probably wouldn’t use it.” (Interviewee 6)

Closely related to time efficiency were user-friendliness (72%, 13/18) and workflow integration (67%, 12/18). Participants described user-friendly AI as intuitive, responsive, non-intrusive, and requiring minimal effort. Seamless integration was equally important: participants reported already working across multiple software systems, and envisioned ideal AI as embedded within existing systems, with results automatically available in PACS and easily incorporated into reports.

When asked what tasks they would like AI to perform, participants identified a range of activities (Table 2).

**Table 2:** Potential applications of AI in radiology identified by participants.

**Potential AI Application**	**n (%)**	**Description**
Drawing attention to key details	9/18 (50%)	Flagging urgent findings or marking abnormalities such as malignant lesions or fractures, particularly useful for subtle or unexpected findings that might otherwise be missed.
Second reader	9/18 (50%)	Using AI to verify findings and increase diagnostic confidence, either after writing a report or in screening settings such as lung lesion detection or mammography.
Comparison	7/18 (39%)	Automating the time-consuming tracking of changes between imaging studies, particularly in patients with advanced oncologic disease or multiple sclerosis involving multiple lesions.
Quantification	7/18 (39%)	Automatically measuring organ or lesion volumes, such as liver volume for surgical planning, prostate volume, or lung nodule and malignant lesion size for longitudinal comparisons.
Workflow optimization	5/18 (28%)	Optimizing scanning orders, scheduling, email replies, and other organizational tasks.
Report writing	4/18 (22%)	Helping generate reports faster or improving clarity and style.
Scanning improvement	3/18 (17%)	Shortening scanning times or improving image quality.
Clinical information summary	3/18 (17%)	Extracting and summarizing relevant clinical information, such as prior treatments or smoking history.

The most commonly mentioned tasks make it clear that radiologists do not want to be fully replaced, as they value many aspects of their work. Instead, they hope AI can assist by handling repetitive or low-value tasks, allowing them to focus on more meaningful and complex work, including interpreting challenging cases and communicating results with colleagues and patients. As one participant noted:

“Assisting with relatively routine, simple tasks to make them quicker and easier helps us to refocus on the clinical part of radiology — interpreting results, discussing them with clinicians, and handling more in-depth, difficult cases — rather than the routine stuff, like nodule detection or lesion size measurements.” (Interviewee 9)

Many participants expressed the view that current AI tools are specialized and focused on specific tasks (50%, 9/18), and that the ideal AI should have a holistic overview (44%, 8/18). For AI to truly replace human readers, radiologists suggested it would need to integrate information beyond a single image, considering patient age, gender, family history, prior studies, and additional modalities such as lab tests or other imaging. Particularly for unsupervised AI, many emphasized the need to detect all possible findings to avoid missing incidental lesions in unexpected locations.

Training data was another important factor, with participants wanting to know the patient population (50%, 9/18), data quality (17%, 3/18), and number of cases used for training (17%, 3/18) — generally favoring larger datasets. They also suggested that algorithms trained on similar populations or in the same healthcare setting would likely perform better, given closer alignment in patient demographics, disease prevalence, and imaging protocols.

Transparency was frequently mentioned as an important factor (50%, 9/18), referring both to knowing who created the AI and how, and to understanding how the program reaches its conclusions and what might cause it to malfunction. However, most participants acknowledged not needing full technical details and recognizing they might not even be capable of fully comprehending the intricacies of AI. Many compared this to their understanding of CT or MRI scanners or computers — a basic working knowledge sufficient to troubleshoot or recognize when something goes wrong, even if the full technical details remain beyond their expertise.

"The comparison with the physics of MRI or CT is a good one — at least if something goes wrong, you have some understanding of what's happening and can identify the problem. Otherwise, it's like, 'I don't know, sometimes it works, sometimes it doesn't,' and that doesn't reflect well on you as a radiologist. Of course, it's too complex to fully comprehend everything, but you should know the basics.” (Interviewee 11)

### 3.3 Developer-related factors

Sixteen (89%) participants highlighted that the institution behind an AI system influences their trust, with a preference for large universities, university hospitals, or non-commercial research entities over private companies. Trust further increased if the system was adopted by reputable hospitals, endorsed by major radiological societies, or held official European certification.

“I do think that we should be careful that the development of tools is free of commercial bias, so it is in the best interest of the patient, and it's ideally done in collaboration between industrial and clinical partners.” (Interviewee 9)

A few (11%, 2/18) participants noted that while a reputable institution may encourage them to try an AI system, their ultimate decision to adopt it would still depend on accuracy and reliability.

“Of course, it looks better if a university hospital sponsors the project, but even small companies with a good product can earn trust through performance. I wouldn’t worry too much about the size of the developer — if the program works well and I have good experience with it, I’d use it just like any other program.” (Interviewee 18)

Evidence of performance was important to 11 (61%) participants, who cited statistical data, large clinical trials, and peer-reviewed publications in reputable journals as trust-enhancing. Clinician involvement was similarly valued (56%, 10/18): participants were more likely to trust AI systems where colleagues — whether field experts, researchers from reputable institutions, or local clinicians — had contributed to training, validation, or local testing, emphasizing that such involvement ensures clinical relevance.

### 3.4 Ethical considerations

Human agency and oversight emerged as the most prominent ethical theme (83%, 15/18). Radiologists consistently emphasized the importance of maintaining control over the diagnostic process and retaining final decision-making authority, expressing a clear preference for AI as a supportive rather than autonomous tool. They stressed that radiology requires context, clinical reasoning, and experience that AI cannot fully replicate, regardless of its accuracy. Several participants also warned against AI-generated results being sent directly to clinicians or patients without radiologist review, cautioning that bypassing professional oversight could cause unnecessary anxiety or unwarranted follow-up procedures.

**Fig. 5:**
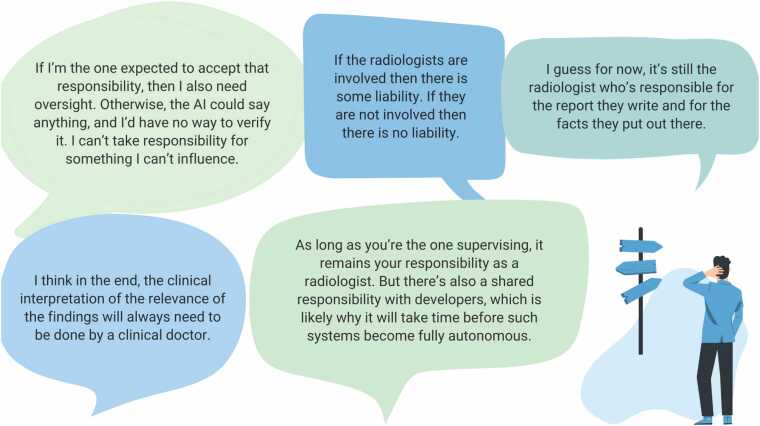
Representative interview excerpts on accountability and responsibility for AI-related errors.

Liability was a frequently discussed ethical concern (Fig. 5). While some participants were uncertain about accountability in cases of AI-related errors (22%, 4/18), most were willing to take full responsibility themselves (50%, 9/18). A smaller group (22%, 4/18) felt accountability should lie with developers or producers, particularly in contexts where AI operates autonomously without human supervision.

Patient privacy (44%, 8/18) and data governance or ownership (33%, 6/18) were also raised as important ethical considerations. Participants emphasized that patient anonymity must be strictly protected and that hospitals contributing data for AI training should retain ownership and control over how that data is used. As one radiologist explained:

“Many of these applications are cloud-based, meaning certain data might be sent to a distant server for processing. That makes it very important to check everything: data security, privacy regulations, making sure we uphold proper standards. In everything we do, we should always ask ourselves: is this benefiting our patients? Are we using their data for their benefit? We need to stay critical of ourselves, especially in diagnostics, working with imaging data. We have to remember — it's patient data.” (Interviewee 12)

### 3.5 Patient-related factors

**Fig. 6:**
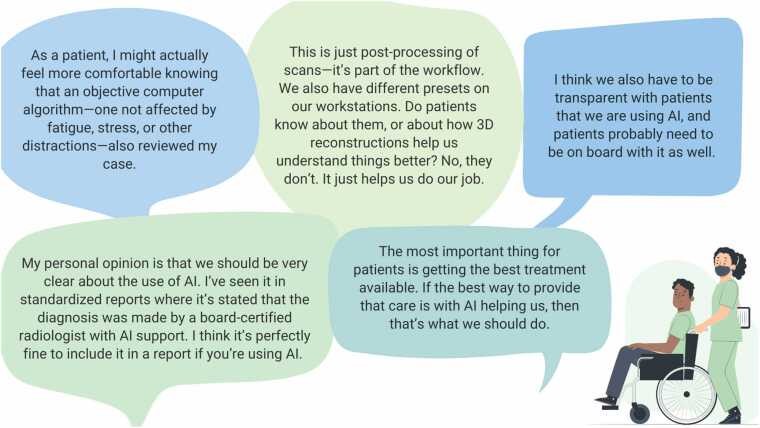
Representative interview excerpts on radiologists’ views of patient perceptions of AI use and the need for disclosure.

Most radiologists believed patients would likely respond positively to AI use, with some anticipating excitement about cutting-edge technology and greater confidence in AI-assisted results. Nevertheless, 10 (56%) participants emphasized that patients should be informed and able to consent, with some proposing opt-in or opt-out options. A smaller group (28%, 5/18), however, felt AI does not need to be disclosed as long as results are reviewed by a radiologist, comparing it to other aspects of imaging patients are typically unaware of, such as post-processing techniques. Their general view was that patients care most about the accuracy of the result, not the tools used to achieve it (Fig. 6).

Two (11%) participants also suggested that greater patient education may be needed in the future to explain AI's role and capabilities and foster broader understanding and trust.

## 4 Discussion

Our study explored the human side of AI adoption in radiology, revealing that trust is shaped not only by technical performance but also by psychological, professional, social, and ethical factors. While most existing research focuses on technical validation, our findings suggest that successful AI integration requires attention to this broader ecosystem of trust [1], [19].

### 4.1 Accuracy, efficiency, and the trade-off between them

Accuracy and reliability emerged as the most consistently cited determinants of trust, consistent with previous reviews [1], [6], [12]. Yet our findings highlight a tension that is less often discussed: accuracy and time-saving, while both highly valued, can pull in opposite directions. A highly accurate AI that introduces delays may be rejected regardless of its diagnostic performance, while a fast but less reliable tool introduces its own risks if trusted uncritically. The acceptable balance may differ considerably depending on clinical context — a finding with practical implications for how hospitals evaluate and procure AI tools [2].

### 4.2 Transparency and its limits

Transparency was frequently cited as important for trust, consistent with recent literature [15], [16], [20]. However, our findings point to an important nuance: participants wanted transparency but acknowledged they might not fully understand technical details even if provided. Rosenbacke et al. (2024) similarly found that explainability does not straightforwardly increase trust and may, in some cases, reduce it. The threshold of transparency needed to foster appropriate trust in different clinical contexts, therefore, deserves further investigation.

### 4.3 Human oversight: principle vs. practice

Radiologists strongly emphasized human oversight as a fundamental safeguard, echoing findings from comparable qualitative work in radiology [13] and broader discussions of liability in clinical AI [11]. Yet this commitment may be difficult to sustain in practice — heavy workloads and time pressure could lead to more superficial review of AI outputs, particularly when the system appears reliable. This creates a paradox: the very conditions that make AI most attractive are also those most likely to undermine oversight quality, increasing the risk of automation bias [8], [9]. Institutional safeguards — such as alerts for high-risk outputs and regular audits of AI-assisted decisions — may be needed to ensure oversight remains substantive.

### 4.4 Current experience vs. future expectations

A recurring pattern was the tendency to blend present-day AI experience with expectations about future capabilities. Concerns about automation bias, deskilling, and job displacement were largely framed as future worries rather than current experiences. This blurring is consistent with observations in the broader AI adoption literature [1] and raises an important methodological question for future research: studies should seek to distinguish trust in currently deployed systems from attitudes toward anticipated future AI.

### 4.5 Deskilling and the future of radiology training

Participants expressed concern that residents trained with constant AI support may fail to develop core diagnostic intuition. This mirrors well-documented concerns in other high-stakes fields about skill degradation following over-reliance on automation [8]. As AI takes over more routine tasks, the radiology community may need to consider how training curricula can preserve fundamental diagnostic skills while supporting effective human–AI collaboration [10], [21].

### 4.6 Trust as a multi-stakeholder phenomenon

Our findings show that trust was shaped heavily by colleagues' opinions, institutional reputation, and developer credibility — not just system performance. This is consistent with research on peer influence in technology adoption [22], [23], [24], suggesting that trust in clinical AI is partly socially constructed and cannot be built through technical improvements alone. In addition, individual and institutional trust do not always align — a radiologist may personally trust an AI tool but still not use it without formal institutional validation [13] - and clinicians and hospitals may weigh the costs, benefits, and risks of AI adoption differently. Patients add a further layer of complexity as a third stakeholder whose acceptance or resistance could ultimately influence whether AI tools are adopted and sustained in practice. This multi-level nature of trust, spanning clinicians, institutions, and patients, deserves greater attention in future research.

### 4.7 Limitations

This study represents an initial attempt to holistically explore the ecosystem of trust in AI within radiology, and findings should be interpreted as exploratory rather than generalizable. The relatively small sample and recruitment from two university hospitals in high-income European countries limit transferability. As all participants had prior AI experience, their views may differ from those of radiologists with limited or no AI exposure or those working in settings with less developed AI infrastructure. Voluntary participation also likely attracted radiologists with a stronger interest in AI, and the cross-sectional design cannot capture how trust evolves over time. The qualitative approach provided depth but did not allow quantitative comparison of the relative importance of trust factors. Future research should examine trust across broader healthcare systems and experience levels, and mixed-method or longitudinal designs could provide more precise measures of how trust develops over time.

## 5 Conclusion

Successful AI adoption in radiology requires more than technical capability — it depends on earning the trust of those who use it. Our findings reveal a broad ecosystem of factors shaping radiologists' trust, spanning system performance, workflow integration, transparency, institutional credibility, and ethical considerations, alongside important tensions between accuracy and efficiency, human oversight and workload, and individual and institutional incentives. Addressing these human factors throughout AI development, implementation, and governance will be essential for creating tools that are not only accurate and reliable but genuinely trusted and widely adopted in clinical practice.

## CRediT authorship contribution statement

**Kalina Chupetlovska:** Writing – review & editing, Writing – original draft, Visualization, Investigation, Data curation, Conceptualization. **Eleni Georganta:** Writing – review & editing, Writing – original draft, Supervision, Project administration, Methodology, Investigation, Conceptualization. **Wouter Dignum:** Writing – review & editing, Data curation. **Saachi Yadav:** Writing – review & editing, Data curation. **Thi Dan Linh Nguyen-Kim:** Writing – review & editing. **Regina Beets-Tan:** Writing – review & editing, Supervision. **Stefano Trebeschi:** Writing – review & editing, Writing – original draft, Supervision, Project administration, Conceptualization.

## Ethical statement

An approval for this study was received from the Ethics Committee of the Faculty of Social and Behavioural Sciences of the Univerity of Amsterdam Amsterdam, Netherlands. This study was conducted in accordance with the Declaration of Helsinki. All participants provided informed consent prior to participation.

## Declaration of Competing Interest

The authors of this manuscript declare no conflicts of interest.
