# Supplementary material for: From resistance to reliance: A human-centered analysis of the spectrum of radiologists' trust in AI
Source: Eur J Radiol Open. 2026 Jun 19;17:100780. doi: 10.1016/j.ejro.2026.100780 (PMC13311287; doi:10.1016/j.ejro.2026.100780)
Supplement: Supplementary file 1 — Supplementary material [file mmc1.docx]

**Interview Guideline**

**Set the Stage**

- Welcome the interviewee, give them some time to make themselves comfortable.
- Introduce yourself (or anyone else that might be attending the meeting).
- Let them know that you might take some notes during the interview or look at your interview guideline to make sure that you will cover all relevant topics.

**Introduction**

1. **Welcome and Purpose**:

- *“Thank you for agreeing to participate in this study. This is a collaborative research project between the University of Amsterdam and the Netherlands Cancer Institute. Our goal is to understand how AI technologies are perceived by radiologists and to identify key factors that ensure AI tools can be trusted when evaluating a tumor's response to treatment. Your insights will play a crucial role in shaping this research.”*

1. **Consent and Confidentiality**:

- *“If you agree, I would like to record our interview to ensure we capture your responses accurately. These recordings will be securely stored on the University of Amsterdam’s server, and all data will be fully anonymized to protect your identity.”*
- *“Please note that your participation is completely voluntary. You can choose to skip any question or stop the interview at any time without providing a reason.”*

1. **Duration**:

- *“The interview will last approximately 45 to 60 minutes. Do you have any questions or concerns before we begin?”*

*(wait for consent and then start recording)*

**Opening**

**Participant Background**:

- “Could you tell me a little about your professional background?”
  - Job title and current role.
  - Length of experience in radiology.
  - Experience working with AI or advanced technologies in medical practice.
- “Have you used AI tools before? How was this experience for you?”

**General Perceptions about AI and technology – Advantages & Disadvantages**

- “Advancements in technologies have impacted many professions, including radiology. What is your general perception of the use of AI in medical imaging or diagnostics?”
- “Which do you think are the most important advantages and disadvantages of AI in radiology?”

**Main Interview Part**

**Theme 1: User-Centric Factors**

**Main Questions:**

- From your perspective, what are your expectations for you to trust AI tools for tumor evaluation? What is relevant for you to trust AI or not to trust it?
- What are the factors that other radiologists might have (e.g., personality, attitudes) that might influence whether they trust or not AI systems in radiology?

**Subquestions**:

- Performance Expectancy: “What do you expect AI to achieve in terms of accuracy and diagnostic support?”
- Effort Expectancy: “How easy or difficult do you find using AI tools? Do they simplify your workflow?”
- Previous Experience: “Have you used AI tools before? How has that shaped your trust in them?”
- General Attitude: “How would you describe your attitude toward AI technologies in general?”
- User Characteristics: “Are there specific standards or personal criteria you apply when evaluating new technologies?”

**Theme 2: System-Related Factors**

**Main Questions**:

- “What characteristics of AI systems do you think are most important to build trust in their use for tumor evaluation?”
- “What characteristics of AI systems do you think might make you or someone else not trust these AI technologies?”

**Subquestions**:

- Reliability & Accuracy: “How important is consistency in performance? What would you consider acceptable margins of error?”
- Transparency: “How important is it for you to understand how AI reaches its conclusions? What level of explanation do you expect?”
- Robustness: “How confident are you in AI’s ability to function under complex or uncertain conditions?”
- Perceived Risk: “What potential risks or negative outcomes do you associate with using AI in your work?”
- “How about their interface, how they communicate information, how they look? Do these factors matter to trust or not AI systems in radiology?” (user-friendliness)

**Theme 3: Designer/Developer-Centric Factors**

**Main Questions**:

- “How does trust in the developers or organizations behind AI tools affect your willingness to use them?”
- “In your opinion, what are the most important factors to trust or not the developer of the AI tool or the company that will see it to your organization?”

**Subquestions**:

- Developer Competence: “Do you think the qualifications and reputation of AI developers influence trust?”
- Clinician Involvement: “Do you believe radiologists should be involved in AI system development? Why or why not?”
- Liability: “How important is it to know who is accountable for errors or inaccuracies from AI tools?”

**Theme 4: Institutional and Ethical Considerations**

**Main Questions**:

- “Do you think that trust in your organization (hospital, institute) matters for trusting an AI system at your work? If so, what aspects are relevant for you that will influence whether you will trust/use this AI system or not?”
- “What other ethical and governance aspects do you think are critical for building trust in AI technologies in healthcare?”

**Subquestions**:

- Human Oversight: “To what extent should radiologists retain decision-making control when using AI?”
- Fairness: “How important is it for AI to avoid biases? Have you encountered concerns in this area?”
- Societal Impact: “How do you see AI contributing to or potentially harming broader societal or environmental goals?”

**Theme 5: The Role of the Patients**

**Main Question**:

- “Patients might also trust or not trust AI. Do you think that this might impact how you trust or distrust AI systems in radiology? Does what the patient thinks or feels matter?”
- “What do you think is important for the patient and consequently for you to trust the AI systems?”

**Subquestions**:

- Privacy: “Do you have concerns about patient data being used by AI systems? What safeguards do you expect?”
- Human Oversight: “To what extent do you think patients would like humans to have the final say and responsibility? What role do you think they believe that AI systems should have in diagnosis?”

**Closing Questions**

1. “If you think about everything that we have discussed that can impact of much we trust AI in radiology, user-characteristics, AI system-characteristics, developer-characteristics, the institution, and the role of the patients, what do **you** think is the most important factor that will helps us trust AI in radiology, and what is the most important factor that will make us not trust it?”
2. “Is there anything else you’d like to share about your experience or expectations regarding AI in radiology?”
3. “Do you have anything else that was not covered in this discussion that you would like to add?”

**Thank You and Wrap-Up**

- *“Thank you so much for your time and valuable insights. Your input will greatly contribute to understanding how AI can be designed and implemented to meet the needs and expectations of radiologists.”*

**General Tips for Interviews**

- *Create a Comfortable Environment:* Help the interviewee feel at ease. Speak calmly and don’t rush so they can relax and open up.
- *Allow Time to Think:* Give them enough time to think and respond, especially since they may not have thought about these questions before. Let them finish their thoughts.
- *Paraphrase for Clarity:* Repeat what they say in your own words to make sure you understood them correctly (e.g., “Do you mean that…?”).
- *Connect to Previous Points:* Mention things they said earlier to show you were listening and make them feel valued.
- *Request Clarifications:* If they aren’t clear, ask follow-up questions or for examples to help them explain their ideas more fully.
- *Adapt to the Conversation Flow:* Change the order of topics if it feels more natural based on what they are talking about.
- *Keep Your Speaking Time Minimal:* Talk only when necessary to summarize, guide the conversation, or ask the next question. Let them do most of the talking.
- *Stay Neutral:* Avoid sharing your opinions about AI. Ask about both what might build or reduce trust in AI without taking sides.
