# Supplementary material for: From resistance to reliance: A human-centered analysis of the spectrum of radiologists' trust in AI
Source: Eur J Radiol Open. 2026 Jun 19;17:100780. doi: 10.1016/j.ejro.2026.100780 (PMC13311287; doi:10.1016/j.ejro.2026.100780)
Supplement: Supplementary file 2 — Supplementary material [file mmc2.docx]

APPENDIX 2 – Code Book

| **1. User-Centric (UC) Factors** | | |
| --- | --- | --- |
| *These factors relate to user characteristics, beliefs, or experiences that influence trust in AI.* | | |
| ***Performance expectancy*** | User’s expectation of how well the AI will perform. | |
| ***Effort expectancy*** | Perceived ease of use and workload reduction. It is coded as positive when the radiologist does not have to manually interfere and it reduces their workload. Negative one is coded as User Burden. | |
|  | ***User burden*** | When AI requires manual input, increasing effort or time; fails to fully automate the task. |
| ***Previous experience*** | Familiarity with AI influences trust. | |
| ***General attitude to AI*** | Interviewee’s overall openness or skepticism toward AI. | |
| ***Colleague’s opinion*** | Whether the colleague’s opinion about a specific AI system also determines the trust/willingness for using it by the interviewee. | |
| ***User characteristics*** | Individual standards or preferences affecting trust. | |
|  | ***Independence vs Assistance*** | Preference for working autonomously vs using AI as support. |
|  | ***Future potential*** | Belief that AI will improve and become more trustworthy in the future. |
|  | ***Automation bias*** | Tendency to over-rely on AI, even when it's incorrect. |
|  | ***Threat*** | Fear that AI might replace the radiologist’s role. |
|  | ***Individual difference*** | Perceived variation in how different radiologists view AI. This is not about the interviewee specifically, but their view of other radiologists. |
|  | ***Technological affinity*** | Comfort and familiarity with technology influencing trust. |
| ***Why****: These factors shape users’ expectations, experiences, and mindset, directly affecting trust in AI.* | | |

| **2. System-Related (SR) Factors** | | |
| --- | --- | --- |
| *These elements describe the inherent characteristics of the AI system that impact trust.* | | |
| ***Technical robustness and safety*** | Ensures the system is reliable and protected against failure. | |
| ***Reliability & Accuracy*** | Consistent and predictable performance. | |
| ***Robustness*** | Ability to function under challenging conditions (e.g., noisy or incomplete data). | |
| ***Performance*** | Effectiveness in completing the intended task. | |
|  | ***Execution time*** | Time required to perform the task. |
| ***Transparency*** | Clarity in how decisions are made and the ability to trace them. | |
| ***Other characteristics*** | General features radiologists find useful. | |
|  | ***User-friendliness*** | Ease of data integration and system functionality. How intuitive and easy-to-navigate the system menus are. |
|  | ***Drawing attention to key details*** | E.g., visual highlights, flagging anomalies. |
|  | ***Workflow integration*** | Integration between systems to reduce effort and time (e.g., cross-compatible hospital servers). |
|  | ***Second reader*** | AI as a second reader/second opinion. |
|  | ***Report writing*** | AI as a support in language editing and report writing/summarizing. |
|  | ***Image quality/speed improvement*** | AI as a tool to reduce time of image acquisition and improve quality of images. |
|  | ***Comparison*** | AI as a tool to automatically compare two exams. |
|  | ***Workflow optimization*** | AI as a tool to optimize schedules and triage. |
|  | ***Quantification*** | Measuring size and volume of lesions automatically. |
| ***(Perceived) ability*** | Whether the AI is seen as capable of performing its task effectively. | |
|  | ***Human-Equivalent AI expectations*** | Whether users believe the AI performs as well as a human radiologist. |
|  | ***Superhuman AI expectations*** | Whether users believe the AI should perform better than a human radiologist. |
| ***(Perceived) risk*** | Concerns about possible negative outcomes of using the system. | |
|  | ***AI dependency (Cognitive offloading)*** | Concern that radiologists would lose their skills (or never develop them in the first place) to think independently . |
|  | ***Hallucination*** | AI augmenting images and creating false findings or obscuring findings. |
| ***Generalized vs specific*** |  | |
|  | ***Generalized*** | Being able to combine knowledge. |
|  | ***Specialized*** | Specific limited task knowledge. |
| ***Holistic overview*** | Ability to view both detailed (micro-level) and broad (macro-level) system perspectives. This is within a single system, not across multiple integrated systems. | |
| ***Training data*** | Data used to develop, test, and update the system. | |
|  | ***Population data*** | Impact of data origin (e.g., limited generalizability if from a single population). Negative when data from a specific country introduces bias, leading to generalization issues. Positive when the population data is diverse and broadly applicable. |
|  | ***Quality of the data*** | Influence of data quality on system performance, especially across regions with differing resources. |
|  | ***Quantity of the data*** | Volume of training data provided to the AI. |
| *Why: These factors focus on the design and operational aspects of the AI, emphasizing how its performance and design transparency build or undermine trust.* | | |

| **3. Designer/Deployer-Centric (DC) Factors** | | |
| --- | --- | --- |
| *These factors concern the creators and implementers of the AI system.* | | |
| ***Developer*** | Trust in the skills and ethical standards of the developers. | |
| ***Institution*** | Trust in the organization or institution behind the AI. Also relates to the certification of systems. | |
|  | ***Morality*** | Ethical values upheld by the institution (e.g., non-commercial goals, transparency). |
| ***Liability*** | Who is seen as legally responsible for the AI’s outcomes. | |
|  | ***Positive*** | Human willingly takes responsibility for AI outcomes. |
|  | ***Negative*** | Responsibility is placed on the AI, especially in case of errors. |
|  | ***Responsibility dilemma*** | Uncertainty over who holds final responsibility—doctor, hospital, or AI. |
| ***Clinicians' involvement in system development*** | Degree to which medical professionals (users/experts) contributed to designing the AI. | |
| ***Reputation of third party*** | How the image/social standing of an institution/individual determines the trust in their manufactured AI system. | |
| ***Evidence/proof of ability*** | Radiologists demanding (independent) evidence-based research from reputable sources to prove the AI’s abilities. | |
| ***Cost*** | Radiologists sharing that high cost might prohibit them from choosing certain AI systems. | |
| *Why: Trust in those who design and deploy AI strongly shapes users' confidence, based on perceived credibility, ethics, and accountability.* | | |

| **4. Ethical Considerations (EC)** | |
| --- | --- |
| *These factors encompass the broader ethical principles that influence how AI is perceived.* | |
| ***Human agency and oversight*** | Ensures users retain control and decisions align with fundamental rights. |
| ***Privacy (patient-related)*** | Ensures that the data collection/use was consented by the patient and that the data is encrypted. |
| ***Data governance/Ownership*** | It is about who owns the data and who manages it. |
| ***Diversity, non-discrimination, and fairness*** | Ensures AI is inclusive and avoids bias. |
| ***Adverse environmental impact*** | Covers concerns about the effect that AI can have on the environment (nature) |
| ***Accountability*** | Covers auditability, minimizing negative impact, and providing redress. |
| ***Societal Impact*** | Social consequences of using/buying such AI systems. |
| *Why: These themes reflect the external policies and ethical considerations that shape trust by ensuring fairness, privacy, and respect for human rights.* | |

| **5. Patient Related (PR) Factors** | |
| --- | --- |
| *These factors relate to whether it matters or not to the radiologists how the patient perceives the AI.* | |
| ***Patient involvement*** | Patients need to be aware of the use of AI and approve of its use. |
| ***Patient’s attribution of responsibility*** | If AI contributes to a mistake, who do patients hold responsible—the radiologist, the AI system, the hospital, or a combination? |
| ***Patient education*** | Patients need to be educated on the benefits and risks of AI. |
| *Why: Patients’ trust and involvement in the decision to use AI or not can impact the radiologist’s willingness as well. Suppose if a patient is okay with AI being used and is well informed, in case of wrong diagnosis, they are less likely to sue the doctors. Which in turn, reduces the responsibility dilemma in radiologists.* | |
