# Supplementary material for: From resistance to reliance: A human-centered analysis of the spectrum of radiologists' trust in AI
Source: Eur J Radiol Open. 2026 Jun 19;17:100780. doi: 10.1016/j.ejro.2026.100780 (PMC13311287; doi:10.1016/j.ejro.2026.100780)
Supplement: Supplementary file 3 — Supplementary material [file mmc3.docx]

APPENDIX 3 - COREQ checklist

The Consolidated Criteria for Reporting Qualitative Studies (COREQ): 32-item

| **No** | **Item** | **Notes** |
| --- | --- | --- |
| **Domain 1: Research team and reflexivity** | | |
| Personal Characteristics | | |
| 1. | Interviewer/  facilitator | KC, EG, and WD conducted the interviews. SY assisted with data collection and coding. |
| 2. | Credentials | KC: MD  EG: PhD |
| 3. | Occupation | KC: Research Fellow in Radiology  EG: Assistant Professor in Work and Organizational Psychology  WD and SY: Research Assistant (Student) |
| 4. | Gender | KC: Female  EG: Female  WD: Male  SY: Female |
| 5. | Experience and training | EG had extensive experience in qualitative research methodology. KC had prior experience with clinical research. KC, WD, and SY received training and supervision from EG before and during data collection, with feedback loops provided after conducting initial interviews and after initial coding to ensure consistency. |
| Relationship with participants | | |
| 6. | Relationship established | Participants from the Netherlands Cancer Institute were familiar with KC through her role as a research fellow. No other members of the research team had any prior professional or personal relationship with participants before the start of the study. |
| 7. | Participant knowledge of the interviewer | Participants were informed that this was an academic research study on trust in AI in radiology conducted by researchers from the Netherlands Cancer Institute and the University of Amsterdam. They had no prior knowledge of the researchers' personal views on AI. |
| 8. | Interviewer characteristics | The research team was interested in human factors and AI adoption in clinical practice. KC brought a clinical radiology perspective and EG expertise in organizational psychology and trust, which informed both the interview guide and the analytical approach. |
| **Domain 2: Study design** | | |
| Theoretical framework | | |
| 9. | Methodological orientation and Theory | The study employed semi-structured interviews analyzed using framework-assisted thematic analysis. Data analysis followed an iterative procedure in which transcripts were first coded deductively using a predefined coding scheme derived from five domains identified in the literature on trust in AI across psychology, human–AI interaction, and radiology (see Appendix XX). |
| Participant selection | | |
| 10. | Sampling | Convenience sampling was used. Participants were recruited from two university hospitals — the Netherlands Cancer Institute (Amsterdam, the Netherlands) and City Hospital Zurich (Zurich, Switzerland). Inclusion criteria required that participants be practicing radiologists or radiologists engaged in research. |
| 11. | Method of approach | Participants were recruited via email. |
| 12. | Sample size | There were 18 participants in the study. |
| 13. | Non-participation | Eighteen of 23 radiologists agreed to participate, resulting in a non-participation rate of approximately 22%. Participation was voluntary, and no incentives were offered. |
| Setting | | |
| 14. | Setting of data collection | Participants from the Netherlands Cancer Institute were interviewed in a meeting room at the hospital. Participants from City Hospital Zurich were interviewed online from their workplace. |
| 15. | Presence of non-participants | There were no people present during the data  collection besides participants and researchers. |
| 16. | Description of sample | Eighteen radiologists (see *Fig. 1* for demographic details), ranging from residents to senior consultants, participated. Most were from the Netherlands or Switzerland. All had prior experience with AI in clinical or research settings. |
| Data collection | | |
| 17. | Interview guide | See the Interview Guide in the *Supplementary material* (Appendix 1). |
| 18. | Repeat interviews | There were no repeat interviews with the same  participants. |
| 19. | Audio/visual recording | All interviews were audio recorded with the permission  of the participants. |
| 20. | Field notes | Interviewers made brief notes during and after each interview to capture initial impressions and contextually relevant observations. |
| 21. | Duration | Interview duration ranged from 23 to 51 minutes (median 30 minutes; mean 32 minutes). |
| 22. | Data saturation | Thematic saturation was monitored throughout the analysis. No substantially new themes emerged in the final interviews, suggesting adequate coverage of the key topics within the sample. |
| 23. | Transcripts returned | Transcripts were not returned to participants for  comment and/or correction. |
| **Domain 3: Analysis and findings** | | |
| Data analysis | | |
| 24. | Number of data coders | Data was coded by two coders (SY and KC) with guidance from EG. |
| 25. | Description of the coding tree | See the Coding Scheme in the *Supplementary Material* (Appendix 2). |
| 26. | Derivation of themes | Themes were derived primarily inductively from the data, with the five predefined interview domains providing a partial deductive structure. Codes were generated from participant responses, grouped into subthemes, and consolidated into higher-order themes through iterative discussion among the research team. |
| 27. | Software | Amberscript (https://www.amberscript.com) and Otter.ai (https://otter.ai) were used to assist with transcription during the study. No other specialized software was used. |
| 28. | Participant checking | Participants did not provide feedback on the findings. |
| Reporting | | |
| 29. | Quotations presented | Selected participant quotations are presented throughout the results section to illustrate key themes. Each quotation is identified by interviewee number to allow traceability while preserving anonymity. Additional quotes are provided in the *Supplementary material* (Appendix 4). |
| 30. | Data and findings consistent | All findings are grounded in the interview data. Themes are supported by illustrative quotations and reflect patterns observed across multiple participants. |
| 31. | Clarity of major themes | Major themes are organized according to the five domains of the analytical framework and are clearly defined and described in the results section. |
| 32. | Clarity of minor themes | Divergent views and minority perspectives are discussed throughout the results section, including cases where individual participants expressed opinions contrary to the prevailing pattern. |
