# Supplementary material for: From resistance to reliance: A human-centered analysis of the spectrum of radiologists' trust in AI
Source: Eur J Radiol Open. 2026 Jun 19;17:100780. doi: 10.1016/j.ejro.2026.100780 (PMC13311287; doi:10.1016/j.ejro.2026.100780)
Supplement: Supplementary file 4 — Supplementary material [file mmc4.docx]

APPENDIX 4 – Additional quotes

**User-centric factors**

- Attitude towards AI and Future potential
  - *From what I've seen until now, it's been generally a positive experience, what we have been able to use. I'm hopeful for solutions that are being developed, that they will make our lives easier.* (Interviewee 1)
  - *I think it can be really useful, and I think it is going to be more useful in the future. But I think it should provide more than it does currently. I believe it can do a lot, and with the technology advancing, it will be able to do more and more. And also to save us time.* (interviewee 4)
  - *I mean, for me, it is interesting to perform research on these projects, and I think it will become better and better. But for now, as I said, it may sometimes be a nice add-on for very specific questions, but often it does not help much at the moment.* (Interviewee 5).
  - *I think it will have a huge impact in the future on our job. I think what is important is that our work becomes easier. Maybe the repetitive tasks, things that take a lot of time but are not really difficult for us, but just take up a lot of our time. I think that is the direction I hope and think we are going in. And then, of course, the sequences will become faster in the future, and everything is moving in that direction as well.* (Interviewee 7)
  - *Well, there are two sides to the coin, right? Every powerful tool can be used for good or for bad. AI is no different—it can be used for good and for evil, because that’s how it is with everything. If you give a human a tool, like a hammer, they can build with it, or they can crush things with it. It’s the same with AI.* (Interviewee 10)
  - *I think I am more optimistic. I will say there are some AI tools which are good to have, but I don't think you really need them. But they are very good possibilities to make things a little bit easier. Like if you have a program which calculates all the lung nodules, I don't have to sit there and search for them (...) I think this is the part where AI can really help. And also, there are AI programs for X-ray that can detect fractures (...) this is very good help for clinicians in the emergency department, because as radiologists we cannot see everything immediately. So this is a very good help to categorize it: is it something or not, and if they are not sure it highlights where to check. So overall, there are a lot of helpful things already.*  (Interviewee 16)
- Colleagues’ opinions
  - *If I know these are respected persons, I would still want to check the tool, but of course, if I know that someone who is really famous in the field and has high accuracy themselves has checked it, it is certainly a recommendation.* (Interviewee 5)
  - *But if an application can really show that it is good and trustworthy, I would also look at whether other people are using it and how it is perceived in the field, for example at congresses or in other professional settings. That would be my approach.* (Interviewee 14)
- Radiologists losing their jobs to AI (AI as a threat)
  - *I don’t think we’re going to get replaced—at least not in the next few years. I’m optimistic that all the work that’s not very interesting, but still has to be done, will be handled by AI. Things like lung nodules, for example, every radiologist will get help from AI with that.* (Interviewee 14)
  - *I think some applications are going to replace many other people before they replace radiologists. Especially for more specialized tasks that require more thinking. I assume we are still far from being replaced.* (Interviewee 14)
- Automation bias and AI dependency
  - *Because we do not trust it enough, we are still used to being critical and checking it. But I do not know how that will evolve in a few years. I think this is a problem with AI in general for humans, because you get used to the information it provides. Sometimes you cannot fully check it. The same can happen with radiologists. We just have to be careful with it.* (Interviewee 2)
  - *And for me, another very important point is automation bias. If we rely too much on an AI algorithm, then we become lazy. We think, okay, the AI will do it. And then we rely on the AI because we have seen that it can do pretty well in some cases. And we stop working in the way we normally would. If the AI can do it, I do not put much effort into reading new information or diving into the patient’s history.* (interviewee 3)
  - *The other potential downside, which I have also experienced myself, is that if an AI algorithm has already ruled out a disease, and you start trusting its high negative predictive value, you might become less careful in reviewing the scan yourself. So it could lead to potential false negative findings for human readers, because they rely so much on the AI that they go through the scan more quickly.*

*If the AI has already told us the scan is negative, we probably will not put as much effort into looking for positive findings ourselves. So there is a potential grey zone and risk area there.* (Interviewee 9)

**System-related factors**

- Accuracy and reliability
  - *I think it is important not to make false positives, especially if they are not easily corrected or supervised by a radiologist. If it wants to be a standalone system, it should not make those mistakes. There should be a level of accuracy similar to a human.* (Interviewee 4)
  - *If you have an MRI or a CT scanner from Siemens, Philips, and GE, the scans are basically the same but still slightly different. The image impression is a bit different, and the scan parameters are a bit different. So if there is an AI that can account for these small differences and still give accurate results, that would of course be the best case. But if that does not work because the scanner is a bit different, then that is not as good as being able to use it with any scanner, any exam, and so on.* (Interviewee 6)
  - *So we first need very good AI tools and models that actually perform at a level that can support our workflows. I think a lot of what we see in the media and in research are publications showing that AI has surpassed the radiologist, but most of the time these results are not transferable to a clinical setting. There is so much heterogeneity in patients and data that the AI tool is probably not providing the same value in that setting as it does in a research paper.* (Interviewee 12)
  - *I think the first thing is that it helps improve sensitivity and specificity. That is the primary goal. I do not think it is realistic to say that it will save us time, at least not until we can use it as a first reader. But as long as it is preventing mistakes, that is already valuable.* (Interviewee 18)
- Execution time, user-friendliness, and workflow integration
  - *The most important thing that would stop me from using it is probably poor implementation. If it did not offer its results in a way that is useful to me and easy to translate into a report or share with someone.* (Interviewee 1)
  - *And also, I would make the app user-friendly because sometimes it might be a problem. Sometimes the performance of the model itself can be really nice, but you just don’t want to use it because you don’t understand where to click. And it’s not fancy, like the colors are ugly. Sometimes that also matters. It’s important for the user to have it implemented into their workflow, as it’s mostly going on their PC or the radiologist’s workstation.* (Interviewee 2)
  - *The second thing is that it has to save you time. So that’s two things. The most important is accuracy. But even if it is accurate, if you have to invest ten minutes per scan, you still will not use it.* (Interviewee 5)
  - *Then I have to wait one minute for the result. So I don’t think the time I spend on that is compensated by the findings from the AI. To be time-efficient, it needs to be quick and preferably integrated into the PACS system.* (Interviewee 4)
  - *It shouldn’t divert you from your normal workflow. It should integrate into it. Whenever it detects something—like an abnormality—it should give a pop-up, change a color, or subtly alert you that something is wrong, without you having to open another program or check manually. That doesn’t work. You just want to keep doing your job, and every now and then have the system say, “Hey, check this one.”* (Interviewee 9)
  - *It’s only logical to use it if it can be implemented directly within your report, or if the report is automatically created. If I have to open another application and enter another password—which then expires after a month and needs to be changed again—it becomes too cumbersome.* (Interviewee 15)
  - *It would be easiest to integrate it into our PACS system, because I don’t want another screen. We’re already working with multiple computer screens to look at CT scans, so I don’t want a phone or a separate device. It needs to be in the PACS system.* (Interviewee 17)
- Specialized versus holistic overview
  - *I mean, most of the solutions we have now that rely on small samples, they're small, like individual research projects for one single question, let's say compare lesions for that in that disease. And then we're far from like put in a CT and get a like a report. (Interviewee 5)*
  - *A lot of them are very, very specific—just one tiny part of the job—so in many cases it feels more like a gimmick than actual help.* (Interviewee 11)
  - *It needs the clinical background. It needs to know whether the patient had surgery, radiotherapy, or chemotherapy, so you can understand that, for example, in a brain lesion, calcification might be post-therapeutic rather than a calcifying tumor. The AI program would need access to all past records and patient information so it can better differentiate and take a more complete view of the patient.* (Interviewee 13)
- Training data
  - *I think it is good to know, for example, with mammography, which I was more involved in, that there are hundreds of cases from different hospitals that are pooled together to generate the dataset. So I know it comes from a large pool with cases from different hospitals. But how exactly it is calculated and how the algorithm works behind the scenes, I am not so much into the technical side. Still, to some extent, I think it is important to know.* (Interviewee 16)
- Transparency
  - *Transparency is very important as well. People should understand that this AI works because it was trained on thousands of CT scans segmented by multiple qualified radiologists. They should also understand that the data was processed carefully, and that it is legal. Educated professionals prepared and segmented this data. Maybe it’s not necessary for people to know the details of the model architecture—especially non-technical people, who might not want to go into that level of detail—but it is important to know about the data. That knowledge makes me trust it more. (Interviewee 2)*
  - *I think it’s pretty essential for a radiologist to know at least the basics of how an image is produced. And the same goes for artificial intelligence—because if you don’t know how the software reached its conclusion, you can’t really be sure how much you can trust it. (Interviewee 11)*
  - *But in the end, I think even the people creating it don’t really know what the computer is calculating inside the convolutional network. So I think it’s important to understand how the data is trained, but I’m not sure how much more information a regular user like me needs to know. It’s also like using computers every day—we don’t know exactly how the GPU or CPU works, and yet it still functions and we accept that. So I’m not sure you really need to know all the details. (Interviewee 15)*

**Designer/deployer-centric factors**

- Institution
  - *I should not be biased about that. I mean, we have seen that countries, universities, teams — it is all about politics, funding, and all these metrics. But still, if the AI performs well, it should not be something that affects the decision.* (Interviewee 3)
  - *The world is so complicated now that many large companies have subsidiaries and work with third-party companies. In the end, nobody really knows where everything is produced. So I would not say that this is my biggest concern. I would rather use a program from an unknown company if it has been shown to perform well and I have had a good experience with it. In that case, I would trust it just as much as any other program.* (Interviewee 15)
- Evidence of performance
  - *Well, there just has to be consistency, or studies that show consistent results. It’s not enough to have one study showing great accuracy and great results for AI. You need to show that there is consistency and that the AI has a clear purpose and that this purpose is being fulfilled regularly. But it has to be tested several times before you can actually trust it in different settings.* (Interviewee 6)
- Clinician involvement
  - *What is critical for training these AI solutions is that the baseline data you put in is checked and correct, otherwise the output will be wrong. And then this again comes down to accuracy. If they do not have the right personnel, for example radiologists, to assess the baseline data, the output will always be incorrect.* (Interviewee 5)
  - *I think clinicians should be involved. And I think they were involved in the programs I use, because of those clinical things that, like I said, you do not want to interfere with workflows, things like that. Developers cannot come up with that themselves. I assume they were involved. I think it is important that, if you want to help the clinician in the first place, you need to know what they need help with and how they would like to get that help.* (Interviewee 8)
  - *I would say it would influence my opinion whether clinicians were involved, because they are the users. And if I know that radiologists who have worked in the field for a long time have used it and think it is a good thing, I would trust it more. I would think, okay, other radiologists are working with it and they like it.* (Interviewee 16)

**Ethical considerations**

- Human agency and oversight
  - *For me personally, I find it very important that I have the final say and that nothing is approved without me or another clinician. As a patient, I would not like software deciding what the test results would be. When results are not binary, like lab results that are either elevated or not, that is simple and a machine can do that. But imaging diagnosis is more variable. If you have ten people look at the same images, you will have nine similar results and one that is slightly different. That matters. You want someone who knows what they are doing to look at it. I would not mind that person being helped by a system, but I would not want the system itself to make the final decision.* (Interviewee 8)
  - *I think the in the end, the clinical interpretation of the relevance of the findings will always need to be done by a clinical doctor, or whether a radiologist or another clinical doctor.* (Interviewee 9)
  - *I would want to be the last person saying yes or no, even if I trust the AI and I check it and agree. I think it is good to still have control, and also responsibility, because it can change a lot of the therapeutic follow-up afterwards. And I think if it is really relevant, for example if I were a patient, I would want a doctor to also check it and not just rely on the AI.* (Interviewee 16)
  - *It depends on how good the system is, of course, but I think you will always need a radiologist to determine whether a finding is relevant for the patient. The system can show you that there is, for example, a one-centimeter nodule. But a radiologist also has to consider other factors, such as the patient’s age and how long the nodule has been present.* (Interviewee 17)
- Liability dilemma
  - *I think because in the end some human being has to accept responsibility for a report. It is usually not the company that produces the AI. And if I have to accept responsibility for something, I also need oversight, because otherwise it could just say anything and I cannot verify it. It’s fair — I don’t think I can accept responsibility for something I cannot influence.* (Interviewee 1)
  - *But I do not think it is my responsibility to double-check whether the AI tool we are using is correct. I think that is the responsibility of the vendor. For example, with the multiple sclerosis tool we use, the first time I saw it, I checked it myself to see how it worked. It was actually better than me. After that, I trusted it completely. So unless it is proven otherwise through research studies, by the manufacturer, or by other users, I would probably trust it.* (Interviewee 15)
- Patient privacy and data governance or ownership
  - *I think we do not want to have an AI algorithm running on a server somewhere else while we are working in this hospital. I think the algorithm should be within the hospital and should not be accessible from outside. That is important for being able to say that it is safe and secure. In our hospital, many patients consent to the use of anonymized data for research. So as long as the data are anonymized, I do not see a problem.* (Interviewee 18)

**Patient-related factors**

- Patient involvement
  - *I think we also have to be transparent with patients that we are using AI. And patients probably need to be on board as well.* (Interviewee 1)
  - *Well, I don’t know how it works legally, whether they are entitled to know what programs have been used in the process of the imaging and the reporting. I’m not sure about that. But I don’t think it is too important for patients to know that.* (Interviewee 7)
  - *I think patients have the right to know and should be informed. From the patient perspective, they just want the best possible treatment, and if that includes AI, at some point they need to be aware and informed. But of course, if it is just another tool in our large toolbox.* (Interviewee 12)
- Patient education
  - *I think it is good for patients to realize that some of these AI tools are very powerful and can improve the diagnostic accuracy of radiologists, so they should also be aware of the benefits of having these tools available. Patient education is also relevant to avoid patients experiencing reluctance or anxiety toward these types of tools, and to help them see how it can be beneficial for their diagnostic process. So I do think that, in addition to embedding AI education as part of clinical training for doctors, there should also be increased patient understanding and awareness of how AI can contribute to their diagnosis and treatment.* (interviewee 9)
